# Spot sputum samples are at least as good as early morning samples for identifying *Mycobacterium tuberculosis*

**DOI:** 10.1186/s12916-017-0947-9

**Published:** 2017-10-27

**Authors:** Michael E. Murphy, Patrick P. J. Phillips, Carl M. Mendel, Emily Bongard, Anna L. C. Bateson, Robert Hunt, Saraswathi Murthy, Kasha P. Singh, Michael Brown, Angela M. Crook, Andrew J. Nunn, Sarah K. Meredith, Marc Lipman, Timothy D. McHugh, Stephen H. Gillespie, On behalf of the REMoxTB Consortium, Andreas Diacon, Madeleine Hanekom, Amour Venter, Rodney Dawson, Kimberley Narunsky, B. Mtafya, N. Elias Ntinginya, Andrea Rachow, Evans Amukoye, B. Miheso, M. Njoroje, Noel Sam, D. Damas, Alphonce Liyoyo, A. Ahmad Mahayiddin, C. Chuchottaworn, J. Boonyasopun, B. Saipan, Shabir Lakhi, D. Chanda, J. Mcyeze, Alexander Pym, N. Ngcobo, Cheryl Louw, H. Veldsman, Gerardo Amaya-Tapia, T. Vejar Aguirre, D. K. Chauhan, R. K. Garg, N. K Jain, A. Aggarwal, M. Mishra, S. Teotia, S. Charalambous, N. Hattidge, L. Pretorious, N. Padayachi, L. Mohapi, M. Gao, X. Li, L. Zhang, Q. Zhang, S. Aggarwal, Ketty Belizaire, Majda Benhayoun, D. Everitt, Ann Ginsberg, Martino Laurenzi, Bridget Rawls, Christopher Ridali, Mel Spigelman, Almarie Uys, Christo van Niekerk, Anna LC Bateson, Matthew Betteridge, S. Birkby, Emily Bongard, Michael Brown, Holly Ciesielczuk, C. Cook, E Cunningham, James Huggett, Robert Hunt, Clare Ling, Marc Lipman, Paul Mee, Michael E Murphy, Saraswathi E Murthy, Felicity M. R. Perrin, Robert Shorten, Kasha P Singh, K. Smith, Victoria Yorke-Edwards, Alimuddin Zumla

**Affiliations:** 1 UCL Centre for Clinical Microbiology, Department of Infection, University College London, Royal Free Campus, Rowland Hill Street, London, NW3 2PF UK; 2 Medical Research Council UK Clinical Trials Unit at University College London, Aviation House, 125 Kingsway, London, WC2B 6NH UK; 3 Global Alliance for Tuberculosis Drug Development, New York, NY 10005 USA; 4 London School of Hygiene and Tropical Medicine, Keppel Street, London, WC1E 7HT UK; 5 UCL Respiratory, Division of Medicine, Royal Free Campus, Rowland Hill Street, London, NW3 2PF UK; 6 School of Medicine, Medical and Biological Sciences, University of St Andrews, North Haugh, St Andrews, KY16 9TF UK

**Keywords:** Tuberculosis, Smear microscopy, Early morning sputum, Spot sputum, Diagnostics

## Abstract

**Background:**

The use of early morning sputum samples (EMS) to diagnose tuberculosis (TB) can result in treatment delay given the need for the patient to return to the clinic with the EMS, increasing the chance of patients being lost during their diagnostic workup. However, there is little evidence to support the superiority of EMS over spot sputum samples. In this new analysis of the REMoxTB study, we compare the diagnostic accuracy of EMS with spot samples for identifying *Mycobacterium tuberculosis* pre- and post-treatment.

**Methods:**

Patients who were smear positive at screening were enrolled into the study. Paired sputum samples (one EMS and one spot) were collected at each trial visit pre- and post-treatment. Microscopy and culture on solid LJ and liquid MGIT media were performed on all samples; those missing corresponding paired results were excluded from the analyses.

**Results:**

Data from 1115 pre- and 2995 post-treatment paired samples from 1931 patients enrolled in the REMoxTB study were analysed. Patients were recruited from South Africa (47%), East Africa (21%), India (20%), Asia (11%), and North America (1%); 70% were male, median age 31 years (IQR 24–41), 139 (7%) co-infected with HIV with a median CD4 cell count of 399 cells/μL (IQR 318–535). Pre-treatment spot samples had a higher yield of positive Ziehl–Neelsen smears (98% vs. 97%, *P* = 0.02) and LJ cultures (87% vs. 82%, *P* = 0.006) than EMS, but there was no difference for positivity by MGIT (93% vs. 95%, *P* = 0.18). Contaminated and false-positive MGIT were found more often with EMS rather than spot samples. Surprisingly, pre-treatment EMS had a higher smear grading and shorter time-to-positivity, by 1 day, than spot samples in MGIT culture (4.5 vs. 5.5 days, *P* < 0.001). There were no differences in time to positivity in pre-treatment LJ culture, or in post-treatment MGIT or LJ cultures. Comparing EMS and spot samples in those with unfavourable outcomes, there were no differences in smear or culture results, and positive results were not detected earlier in Kaplan–Meier analyses in either EMS or spot samples.

**Conclusions:**

Our data do not support the hypothesis that EMS samples are superior to spot sputum samples in a clinical trial of patients with smear positive pulmonary TB. Observed small differences in mycobacterial burden are of uncertain significance and EMS samples do not detect post-treatment positives any sooner than spot samples.

**Electronic supplementary material:**

The online version of this article (doi:10.1186/s12916-017-0947-9) contains supplementary material, which is available to authorized users.

## Background

In resource-limited areas, travel time and costs associated with accessing healthcare facilities can be a considerable burden to patients being investigated for tuberculosis (TB) and their families [1–3]. The financial burden can equal several months’ salary and may therefore exacerbate or push people into poverty [4, 5]. These costs apply equally to patients found to have TB, and to the vast majority who have an alternative cause for their symptoms. Given 50 million smear investigations for TB are undertaken every year [6], this may have powerful consequences for global health. Furthermore, a substantial proportion of patients are ‘lost’ during the diagnostic pathway and fail to commence TB treatment [7–10], risking poorer treatment outcomes and presenting a continuing reservoir for transmission of TB in the community.

The diagnosis of TB is largely based on smear microscopy of expectorated sputum samples, and will likely continue to be so in those resource-limited settings unable to afford the roll-out of Xpert MTB/RIF (Cepheid, Sunnyvale, CA, USA) and for whom the World Health Organization (WHO) highlights the critical need for ensuring quality of microscopy networks [11]. Early morning sputum samples (EMS) are generally considered to yield a greater number of positive results than spot samples, and to have higher sensitivity and specificity for culture, yet the published data to support this assumption is scarce. Factors that may influence the results of TB smear microscopy include the volume and quality of the sputum sample collected, the time to processing and transport conditions, and the proficiency of the microscopist.

Routine practice in most National TB Programmes has involved collecting three serial sputum samples, termed a spot-morning-spot method, on the basis of a paper by Andrews et al. published in 1959 [12], involving a spot sample collected at the first clinic visit, an EMS brought by the patient to their second visit, and a third spot sample collected at this visit. Later, in 2007, the WHO changed their advice to ‘spot-morning’ on the basis that 95–98% of positive cultures were detected using the first two smears. Either practice commits the patient to attend at least two clinic visits before a diagnosis of TB can be made. The requirement for patients to provide an EMS may prolong the diagnostic pathway and risks losing patients to follow up. In 2011, WHO advice was revised with a recommendation of a two ‘spot-spot’ strategy collected on the same day; in their guidance document, they quote a 2.8% reduction (95% CI –5.2% to 0.3%) in sensitivity using spot samples [13]. This advice has yet to be implemented widely and only applies to specific settings, emphasizing a responsibility to assure the external quality assurance scheme. Patients providing spot-spot samples rather than awaiting EMS were shown to be less likely to be lost in the diagnostic pathway (2% vs. 5.8%), suggesting a trade-off needs to be made between maximising sensitivity and keeping people in the diagnostic pathway.

In addition to their diagnostic value, qualitative and quantitative results of sputum smears and cultures are often used as a biomarker of treatment response in the clinic and in clinical trials assessing new anti-tuberculous drugs. Yet, to date, there are no published studies on the effect of using EMS or spot samples for these purposes. Furthermore, current evidence tends to predate the introduction of fully automated liquid culture systems that are being rolled out globally.

Herein, we aim to compare the value of EMS and spot sputum samples and hypothesize that early morning and spot sputum are clinically equivalent in terms of positive yield, their sensitivity for culture in solid and liquid media, and measures of mycobacterial load in a large, well-characterised group of patients being treated for TB as part of the REMoxTB study.

## Methods

We undertook an analysis of all sputum sample results from patients enrolled in the REMoxTB study (Clinicaltrials.gov NCT00864383), which has been described previously [14, 15]. Patients were screened for recruitment if at least one sputum sample was positive for acid-fast bacilli using local laboratory procedures. As part of the screening and enrolment procedures, patients provided two additional pre-treatment samples – usually one spot sample at the screening visit and one early morning sample at the second clinic attendance prior to starting treatment. While on treatment, patients provided one sputum sample, either EMS or spot, at each study visit. After treatment, patients attended the clinic every 3 months for 1 year after completion and were asked to provide one EMS on the morning of their clinic visit with a spot sample collected during their clinic visit. Only paired EMS and spot samples with results in both were analysed.

Patients testing HIV positive during screening were excluded if they were already on anti-retroviral therapy and/or had a CD4 count of less than 250 cells/μL. We excluded data from those patients who did not enrol in the trial.

### Microbiology

The EMS was defined as the first sputum produced by the patient at home on the first urge to cough after waking. If more than 1 h was to elapse prior to attendance at the study clinic, patients were advised to refrigerate the sample or store it in a cool dark place.

For spot samples, study staff coached patients unable to spontaneously expectorate quality samples to take several deep breaths, hold their breath for a moment, and repeat this several times until coughing was induced. They would then cough deeply and vigorously whilst breathing out. Some patients were not able to provide requested sputum samples at every visit. Samples were maintained at 2–8 °C until processing.

Laboratory staff were aware that patients were enrolled in the REMoxTB study but were blind to the treatment allocation and clinical condition of the patient or were only provided minimal data such as that available on a microbiology request form. Laboratory staff requested repeat spot samples if the sample provided, either EMS or spot, was considered of insufficient volume (<2 mL) or of poor quality (e.g. salivary sample); the repeat sample was included as a spot sample for the purposes of these analyses.

Sputum type was recorded in the source documents and the case report form. EMS and spot samples were processed together in batches, as described in the laboratory manual [16]. Briefly, sputum specimens were liquefied with N-acetyl-cysteine and sodium citrate, and decontaminated with sodium hydroxide. The decontaminated sample was used to make a smear and inoculate both a solid Lowenstein–Jensen (LJ) slope and a Mycobacteria Growth Indicator Tube (MGIT) containing liquid media (Bactec 960, Becton Dickinson, USA). Smears were stained using a standard Ziehl–Neelsen (ZN) staining procedure and graded (negative, 1+, 2+, 3+, or 4+) as per American Thoracic Society guidelines [9]. LJ slopes were observed weekly; the week of growth up to week 8 was recorded as a measure of mycobacterial quantification. For liquid culture, the MGIT time-to-positivity (TTP) up to 42 days was recorded. ZN staining was used to confirm the presence or absence of acid-fast bacilli in cultures in solid and/or liquid media showing growth. Contamination was excluded by the absence of growth on blood agar plates. Samples flagging positive in the MGIT that were ZN-negative with no contamination on blood agar were considered false positives. At least one pre- or early treatment culture was confirmed as *Mycobacterium tuberculosis* complex by use of Accuprobe (Gen-Probe, USA).

### Statistical analysis

Results on paired EMS and spot samples collected at a single visit were analysed according to stage of treatment, namely samples collected prior to commencing treatment (pre-treatment) and samples collected at trimonthly visits in the 12 months after completion of study drug (post-treatment). Positive yield in smear and liquid and solid culture, and the sensitivity and specificity of smear for culture, were calculated for EMS and spot samples. Sensitivity and specificity were also calculated for the maximum positive yield of available results, where a positive culture in either or both media was considered positive.

The statistical significance of differences in distributions of smear and smear grading, and culture results, was determined using the Stuart–Maxwell test of marginal homogeneity and χ^2^ test. Agreement between results on EMS and spot samples is described for both binary positive/negative results, and for all results including contaminated and false positive (liquid culture) results. Time to detect a positive culture was used as an inverse measure of mycobacterial burden. Time to detection (TTD) in culture on LJ (LJ TTD) and in MGIT (MGIT TTP) in days were recorded; a TTD of 9 weeks, i.e. 63 days, and a TTP of 43 days was used for negative cultures on LJ and MGIT, respectively. Differences between EMS and spot samples were compared using Wilcoxon signed-rank test for paired samples. Odds ratios were calculated to determine the association between quantitative measures of mycobacterial burden in EMS and spot sputum samples (smear grading, TTD of positive cultures) and unfavourable outcome of TB treatment defined as the combined failure of bacteriological cure and relapse within 1 year of completion of therapy as defined by culture using solid media. As TTD of a positive culture is positively skewed, these values were logarithmically transformed for logistic regression analyses.

Kaplan–Meier plots of time to first positive culture after the completion of 6 months of the study drug were generated and EMS and spot sample results compared using a hazard ratio and the Mantel–Cox log-rank test.

An extract of the prospectively collected REMoxTB database was collated for the purposes of this study after completion of the study [15], and analysed using GraphPad PRISM and Stata 14.

## Results

**Fig. 1:**
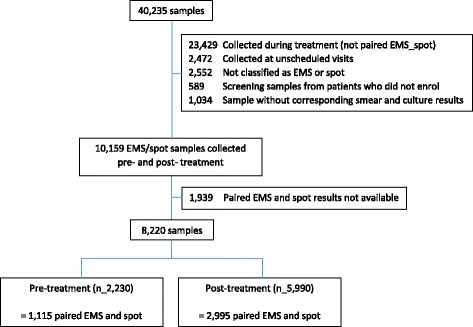
Flow chart of samples included in the EMS and spot study

A total of 1115 pre-treatment and 2995 post-treatment paired spot and EMS samples from 1931 enrolled patients were available for analysis (Fig. 1). Patients were enrolled between 2008 and 2012 from local clinics in South Africa (47%), East Africa (21%), India (20%), Asia (11%) and 1% North America. Males comprised 70% of the population. The median age was 31 years (IQR 24–41). HIV co-infection was detected in 139 (7%) patients; median CD4 cell count was 399 cells/μL (IQR 318–535). A table of full baseline characteristics for the REMoxTB study patient population is available elsewhere [13].

### Smear results

#### Pre-treatment

Prior to treatment, spot samples were smear positive more often than EMS (98.4% vs. 97.0%, *P* = 0.02) as shown in Table 1. Agreement between spot and EMS samples for binary positive/negative results was 95.5%. Somewhat paradoxically, of 1064 positive smears with a smear grading available, 149 (14.0%) had a higher smear grading on the spot sample than the EMS, compared to 409 (38.4%) that had a higher smear grading on EMS compared to spot samples (*P* < 0.001).

**Table 1:** Comparison of paired EMS-spot samples collected pre- and post-treatment

	Paired pre-treatment samples (*n* = 1115)							Paired post-treatment samples (*n* = 2995)				
Smear microscopy			Spot						Spot			
			Negative	Positive	Total				Negative	Positive	Total	
	EMS	Negative	1 (0.1%)	33 (3.0%)	34 (3.0%)		EMS	Negative	2853 (95.3%)	15 (0.5%)	2868 (95.8%)	
		Positive	17 (1.5%)	1064 (95.4%)	1081 (97.0%)			Positive	24 (0.8%)	103 (3.4%)	127 (4.2%)	
		Total	18 (1.6%)	1097 (98.4%)	1115			Total	2887 (96.4%)	118 (3.9%)	2995	
					*P* = 0.02						*P* = 0.15	
Smear grading		Agreement (both positive)			506 (47.6%)			Agreement (both positive)			71 (68.9%)	
		Grading higher on EMS			409 (38.4%)			Grading higher on EMS			24 (23.3%)	
		Grading higher on spot			149 (14.0%)	χ^2^ *P* < 0.001		Grading higher on spot			8 (7.8%)	χ^2^ *P* = 0.002
		Total (where both positive)			1064			Total (where both positive)			103	
LJ culture			Spot						Spot			
			Negative	Positive	Contaminated	Total			Negative	Positive	Contaminated	Total
	EMS	Negative	18 (1.6%)	48 (4.3%)	10 (0.9%)	76 (6.8%)	EMS	Negative	2129 (71.1%)	52 (1.7%)	247 (8.2%)	2428 (81.1%)
		Positive	30 (2.7%)	826 (74.1%)	61 (5.5%)	917 (82.2%)		Positive	31 (1.0%)	85 (2.8%)	11 (0.4%)	127 (4.2%)
		Contaminated	8 (0.7%)	91 (8.2%)	23 (2.1%)	122 (10.9%)		Contaminated	268 (8.9%)	10 (0.3%)	162 (5.4%)	440 (14.7%)
		Total	56 (5.0%)	965 (86.5%)	94 (8.4%)	1115		Total	2428 (81.1%)	147 (4.9%)	420 (14.0%)	2995
						*P* = 0.006						*P* = 0.11
MGIT culture			Spot						Spot			
			Negative	Positive	Contaminated/false positive	Total			Negative	Positive	Contaminated/false positive	Total
	EMS	Negative	2 (0.2%)	12 (1.1%)	1 (0.1%)	15 (1.3%)	EMS	Negative	1979 (66.1%)	67 (2.2%)	227 (7.6%)	2273 (75.9%)
		Positive	9 (0.8%)	998 (89.5%)	34 (3.0%)	1041 (93.4%)		Positive	73 (2.4%)	119 (4.0%)	36 (1.2%)	228 (7.6%)
		Contaminated/false positive	3 (0.3%)	49 (4.4%)	7 (0.6%)	59 (5.5%)		Contaminated/false positive	288 (9.6%)	46 (1.5%)	160 (5.3%)	494 (16.5%)
		Total	14 (1.3%)	1059 (95.0%)	42 (3.8%)	1115		Total	2340 (78.1%)	232 (7.7%)	423 (14.1%)	2995
						*P* = 0.18						*P* = 0.01

*EMS* early morning sputum sample, *LJ* Lowenstein–Jensen, *MGIT* mycobacteria growth indicator tube

#### Post-treatment

Spot and EMS samples had comparable proportions of smear positive rates post-treatment (3.9% vs. 4.2%, *P* = 0.15; Table 1). For the binary positive or negative smear result post-treatment, spot and EMS sample results agreed in 98.7% of cases. Of 103 paired positive post-treatment smears, 8 (7.8%) had a higher smear grading on spot samples compared to 24 (23.3%), which had higher smear grading on EMS (*P* = 0.002).

### Culture results

#### Pre-treatment

**Fig. 2:**
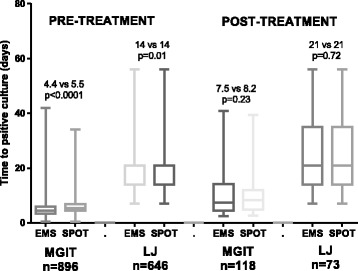
Time to detect positive MGIT and LJ cultures in paired EMS and spot sputum samples collected pre- and post-treatment. Only paired samples where both yielded positive cultures (time to positive culture < 43 days, MGIT and < 63 days, LJ) were included in this analysis. Label in figure shows median and *P* value from Wilcoxon signed rank test

Comparing 1115 paired spot-EMS pre-treatment samples, spot samples were positive more often than EMS in LJ culture (86.5% vs. 82.2%, *P* = 0.006; Table 1). In MGIT culture, both spot and EMS samples had similar positive yields (95.0% and 93.4%, *P* = 0.18). Agreement between spot and EMS samples for any culture result (positive, negative, contaminated (including MGIT false positive)) occurred in 77.8% and 90.3% of cases for LJ and MGIT, respectively. Ignoring contaminated and MGIT false positive results, agreement was of 91.5% and 97.9% for binary positive or negative results for LJ and MGIT, respectively. Agreement between spot and EMS samples for the maximum positive yield of culture results, where either positive in MGIT or LJ was considered positive, was 97.0%. Where paired spot and EMS samples were both culture positive (n = 896), spot samples had longer times to detect a positive MGIT culture than EMS samples by 1 day (median 5.5 vs. 4.5 days, *P* < 0.001), and while the median LJ TTD was 14 days for both spot and EMS samples, the significant *P* value provides evidence for a similar difference (*P* = 0.01, Fig. 2).

#### Post-treatment

Post-treatment, spot and EMS samples had comparable positive yields of culture positives in MGIT (7.7% vs. 7.6, *P* = 0.85) and LJ (4.5% and 5%, respectively, *P* = 0.22). Agreement between spot and EMS samples was 96.4% and 93.7% for binary positive or negative results for LJ and MGIT, respectively. For any culture result, including contaminated and MGIT false-positive results, agreement was 79.3% and 75.4% for LJ and MGIT, respectively. Agreement between spot and EMS samples for the maximum positive yield of culture results, where either positive in MGIT or LJ was considered positive, was 91.0%. Where paired spot and EMS samples were both culture positive (n = 118), there was no evidence of a difference in TTP in MGIT, although the observed TTP in MGIT of spot samples was slightly greater (8.23 vs. 7.5 days, *P* = 0.23; Fig. 2). There were 73 paired spot and EMS sample LJ culture positives, also with no difference in TTD (both 21 days, *P* = 0.72; Fig. 2).

### Contamination rates

Pre-treatment, LJ culture contamination was lower in spot samples than EMS (8.4% vs. 10.9%, *P* = 0.006). There was no difference in the combined MGIT contamination and false positive rate for spot and EMS samples (3.8% vs. 5.5%, respectively, *P* = 0.18). Fewer contaminated spot samples were positive on EMS than were contaminated EMS samples positive on the corresponding spot sample on LJ culture (5.5% vs. 8.2%, *P* = 0.006), but there was no significant difference for MGIT (3.0% vs. 4.4%, *P* = 0.18).

Post-treatment, the combined MGIT culture contamination and false positive rate was lower in spot samples compared to EMS (14.1% vs. 16.5%, *P* = 0.01) but there was no difference for LJ culture (14.0% and 14.7%, *P* = 0.11). Of the contaminated samples on LJ, similar numbers were positive in spot and EMS samples (0.4% and 0.3%), as were contaminated samples in MGIT (1.2% vs. 1.5%).

### Predicting outcomes

**Fig. 3:**
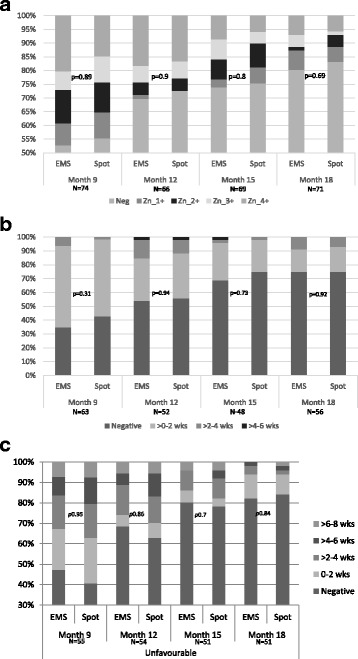
Comparison of paired post-treatment EMS and spot samples in those with an unfavourable outcome; **a** ZN smear; **b** MGIT culture; **c** LJ culture

Of the entire 1931 patients enrolled in the REMoxTB study, unfavourable outcomes were documented in 226 patients (12%) [14]. There was evidence that increasing pre-treatment smear grading predicted an unfavourable outcome in the per protocol population for both spot samples and EMS (non-parametric test for trend; *P* = 0.002 and *P* = 0.008, respectively; Table 2). The increased odds of an unfavourable outcome corresponding to an increasing smear grading for both spot samples and EMS remained significant even when controlling for treatment allocation. However, there was no significant difference in post-treatment-paired spot-EMS smear results and grading, MGIT results and TTP, and LJ results and TTD at any visit both in patients with a favourable and unfavourable outcome (Fig. 3).

**Table 2:** Odds ratios of paired EMS and spot sputum smear grading and culture time-to-detection for predicting an unfavourable outcome (logistic regression)

Baseline predictor variable		EMS			Spot		
		OR	95% CI	*P* value	OR	95%	*P* value
Baseline smear grading*	ZN Neg	0.63	0.18–2.11	0.45	–	–	–
	ZN 1+	0.50	0.17–1.42	0.19	0.22	0.08–0.63	0.004
	ZN 2+	0.38	0.17–0.87	0.02	0.82	0.48–1.39	0.46
	ZN 3+	0.56	0.33–0.95	0.03	0.68	0.43–1.07	0.09
	ZN 4+	Reference			Reference		
Log_10_ LJ TTD		0.61	0.26–1.45	0.26	0.73	0.29–1.87	0.51
Log_10_ MGIT TTP		0.38	0.13–1.10	0.07	0.29	0.08–0.99	0.05

*Unfavourable outcome is associated with increasing ZN smear for both EMS (*P* = 0.008) and spot (*P* = 0.002), non-parametric test for trend

*CI* confidence interval, *EMS* early morning sputum sample, *LJ* Lowenstein–Jensen, *MGIT* mycobacteria growth indicator tube, *OR* odds ratio, *TTD* time-to-detection, *TTP* time-to-positivity, *ZN* Ziehl–Neelsen

Using TTP in pre-treatment samples (an inverse measure of bacterial burden) to predict an unfavourable outcome appeared to be significant for spot MGIT samples (*P* = 0.05) but not EMS (*P* = 0.07) (Table 2). The result for MGIT samples was no longer significant when treatment allocation was included in the model. TTD of a positive culture on LJ media pre-treatment was not predictive of unfavourable outcome for either EMS or spot samples (OR 0.61, *P* = 0.26 and OR 0.73, *P* = 0.51, respectively; Table 2).

**Fig. 4:**
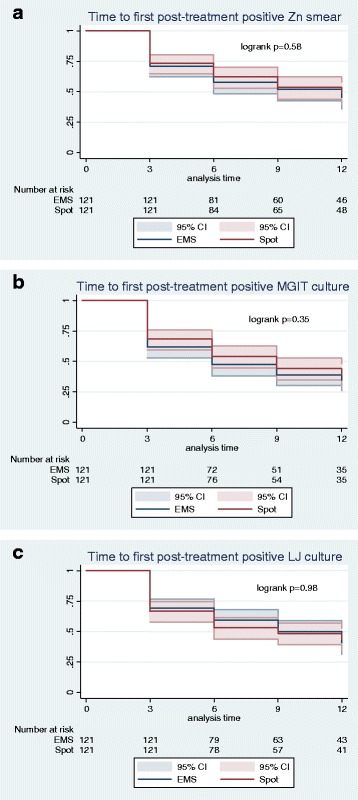
Kaplan–Meier estimates of the time to first positive result in paired EMS and spot samples collected post-treatment for **a** ZN smear, **b** MGIT culture and **c** LJ culture

In those patients with an unfavourable outcome, post-treatment positives were not identified sooner on spot or EMS samples in smear (HR 1.09, *P* = 0.59), MGIT (HR 1.14, *P* = 0.35) or LJ (HR 0.99, *P* = 0.99) (Fig. 4). Comparing paired spot-EMS sputum post-treatment in those with an unfavourable outcome, there was no significant difference in either MGIT TTP or LJ TTD at any patient visit (Fig. 3).

## Discussion

Diagnostic samples collected prior to treatment comprise the greatest proportion of those processed globally for TB and have been the focus of most research in efforts to improve early case detection using smear microscopy. In our study, pre-treatment spot samples had a higher yield of smear positives, 98.4% versus 97.0%, and greater sensitivity for culture in either solid or liquid media compared to EMS, thus supporting spot samples over EMS for the purpose of TB diagnosis. EMS collection adds cost and complexity to patients and health services and may contribute to the considerable drop-out of patients in the diagnostic pathway [7–10]. This strategy may compromise individual patients and risks increased TB transmission in the community, with no useful benefit in terms of case detection.

The time to detect a positive culture in MGIT is inversely correlated with bacterial load [17, 18] and this measure has been used to monitor treatment response and to guide treatment decisions [19, 20]. In pre-treatment samples in our study, we found EMS had faster times to detection in MGIT by approximately 1 day prior to treatment, perhaps owing to pre-culturing in the sample container prior to submission in the clinic as compared to spot samples [17, 21, 22]. Such a small difference is unlikely to be clinically significant. However, this difference may impact the results of molecular identification methods that are used to confirm both the presence of *M. tuberculosis* complex and genotypic resistance patterns, as these have an operational limit for detection and interpretation. As Xpert MTB/RIF (Cepheid, Sunnyvale, CA, USA) is increasingly being considered as a predictor of treatment outcome, and for diagnostic use in resource-limited settings, this difference may indeed prove important [23, 24].

We are not able to address the value of EMS compared to spot samples to identify a positive TB culture in smear-negative patients as smear positivity was an inclusion criterion. The importance of this difference, if any, is limited to those programmatic settings where cultures are used, as opposed to the majority, which rely on smears. Several studies in different settings have shown a higher yield for EMS over spot samples [25, 26]. In contrast, a sub-analysis of a large multicentre study of spot-spot-EMS versus spot-EMS-spot samples processed in solid media (either LJ medium or Ogawa medium) in more than 6000 patients found that spot-spot sample collection alone was not inferior to spot-spot-EMS and indeed resulted in higher numbers of patients actually providing the requested samples [6]. A separate study comparing spot-spot-EMS compared to making two smears from a single sample found them to have comparable sensitivity and specificity for culture-positive TB [27]. Importantly, in both of these studies, and in ours where required, patients were coached on how to provide a quality sputum sample, an intervention which has already proven efficient in improving sputum quality provided by women and is likely to be achievable in programmatic settings [28]. A systematic review and meta-analysis of front-loaded or same-day microscopy compared to standard smear microscopy schedules showed same-day samples to have comparable accuracy for culture-positive TB [29]. These studies are likely to have been influential in the WHO policy statement supporting spot-spot collections [13], and are consistent with our results. WHO guidance supports the use of direct smears in contrast to the decontaminated samples used in our study; however, this is unlikely to have a significant impact given their review of studies and a systematic review of bleach processing failed to demonstrate superiority over direct smears [30, 31].

In our post-treatment samples spot and EMS also had comparable yields of positive smears and cultures on both LJ and MGIT media. Thus, the possibility that EMS identify patients relapsing post-treatment faster than spot samples is not supported by the data in our study. For patients being followed up for relapse in a programmatic setting, these data suggest that a spot sample taken at the clinic visit suffices, with no benefit in awaiting EMS for isolating *M. tuberculosis*.

In terms of predicting outcomes in our study, we identified a trend towards worse outcomes in patients with higher mycobacterial burden on smear microscopy and in MGIT culture, but this finding in MGIT was not significant when adjusted for treatment allocation. There was no association with time to detect a positive LJ culture for either EMS or spot samples.

Microbiological culture data are routinely lost through culture contamination and MGIT false positives. Clearly, there is a balance to be struck between TB isolation and culture contamination; stringent efforts to avoid contamination may reduce the sensitivity of sputum culture by any method. In our study, contamination rates were controlled at between 3% and 8%. Little is known of the reasons for MGIT false positives and thus practical advice to reduce these is unavailable. In our study, spot samples tended to have lower contamination in both MGIT and LJ and lower MGIT false positive rates than EMS both pre- and post-treatment. Our results agree with a study in Ugandan adolescents that found more contamination in EMS [26]. However, they identified non-tuberculous mycobacterial contamination to be an issue, whereas in our study this was mainly with organisms identified on blood agar. Other reported work in this area tends not to be stratified by spot samples or EMS, and specific studies of spot samples and EMS generally remove contaminated samples from their analyses. Hence, contamination differences between spot samples and EMS may have been previously overlooked.

## Conclusions

In this study of patients with smear-positive TB, spot samples were found to be at least as good as EMS for identifying *M. tuberculosis* prior to and during TB treatment and do not support the superiority of EMS over spot samples in a clinical trial setting. This study provides further support for the same-day, two-sample spot-spot diagnostic process recently endorsed by the WHO for programmatic settings. A strategy of no longer requiring EMS collections could have an important impact on global health and may avoid potentially catastrophic costs for individual patients and their families when being assessed for TB.

## Additional file

Additional file 1: List of ethics committee approving the REMoxTB study. (DOCX 893 kb)

## Abbreviations

EMS: early morning sputum sample
IQR: interquartile range
LJ: Lowenstein–Jensen
MGIT: Mycobacteria Growth Indicator Tube
TB: tuberculosis
TTD: time-to-detection (LJ)
TTP: time-to-positivity (MGIT)
WHO: World Health Organization
