# Supplementary figures and images for: Spot sputum samples are at least as good as early morning samples for identifying Mycobacterium tuberculosis
Source: BMC Med. 2017 Oct 27;15:192. doi: 10.1186/s12916-017-0947-9 (PMC5658986; doi:10.1186/s12916-017-0947-9)

Additional file 1 List of ethics committee approving the REMoxTB study


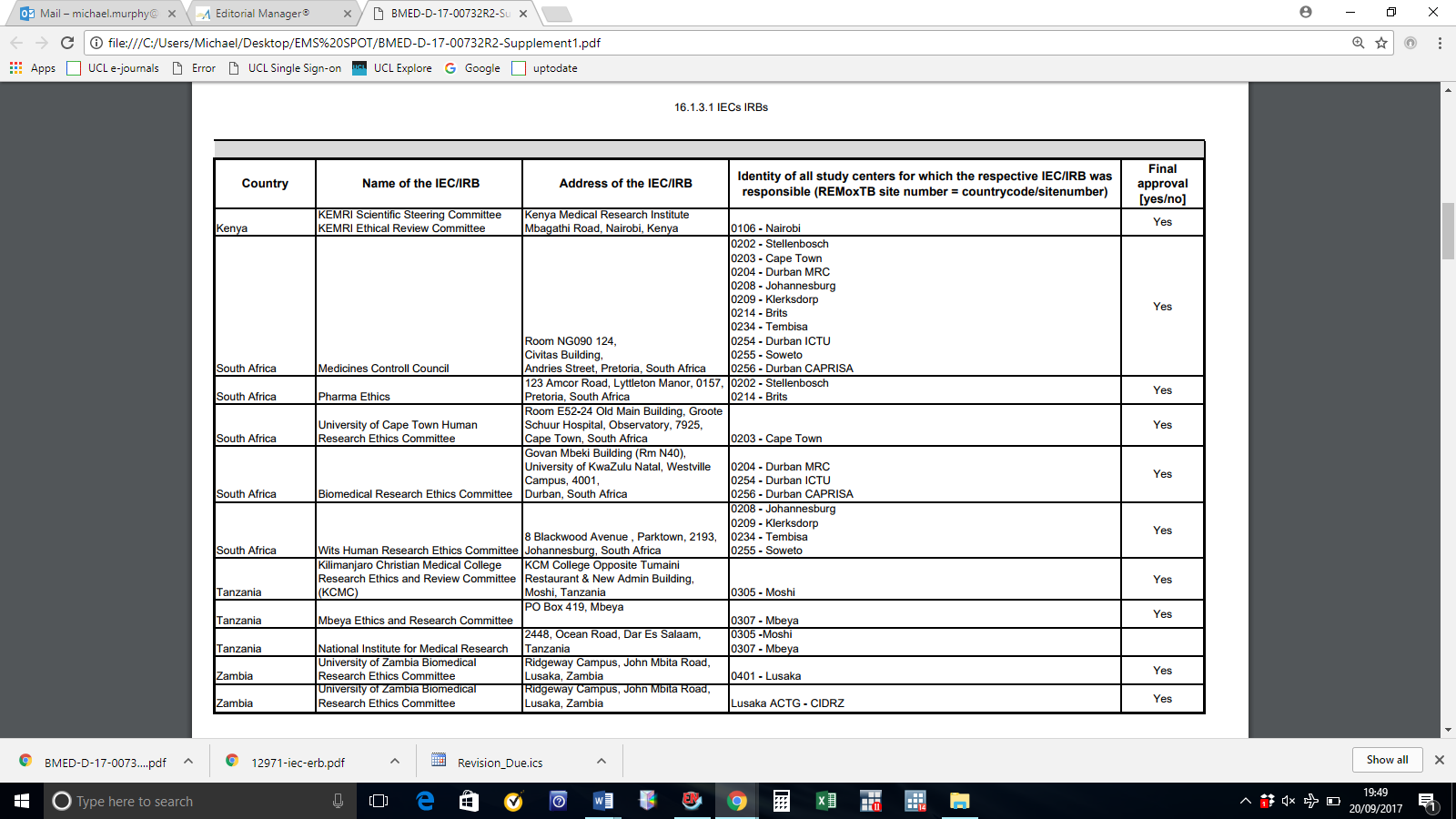


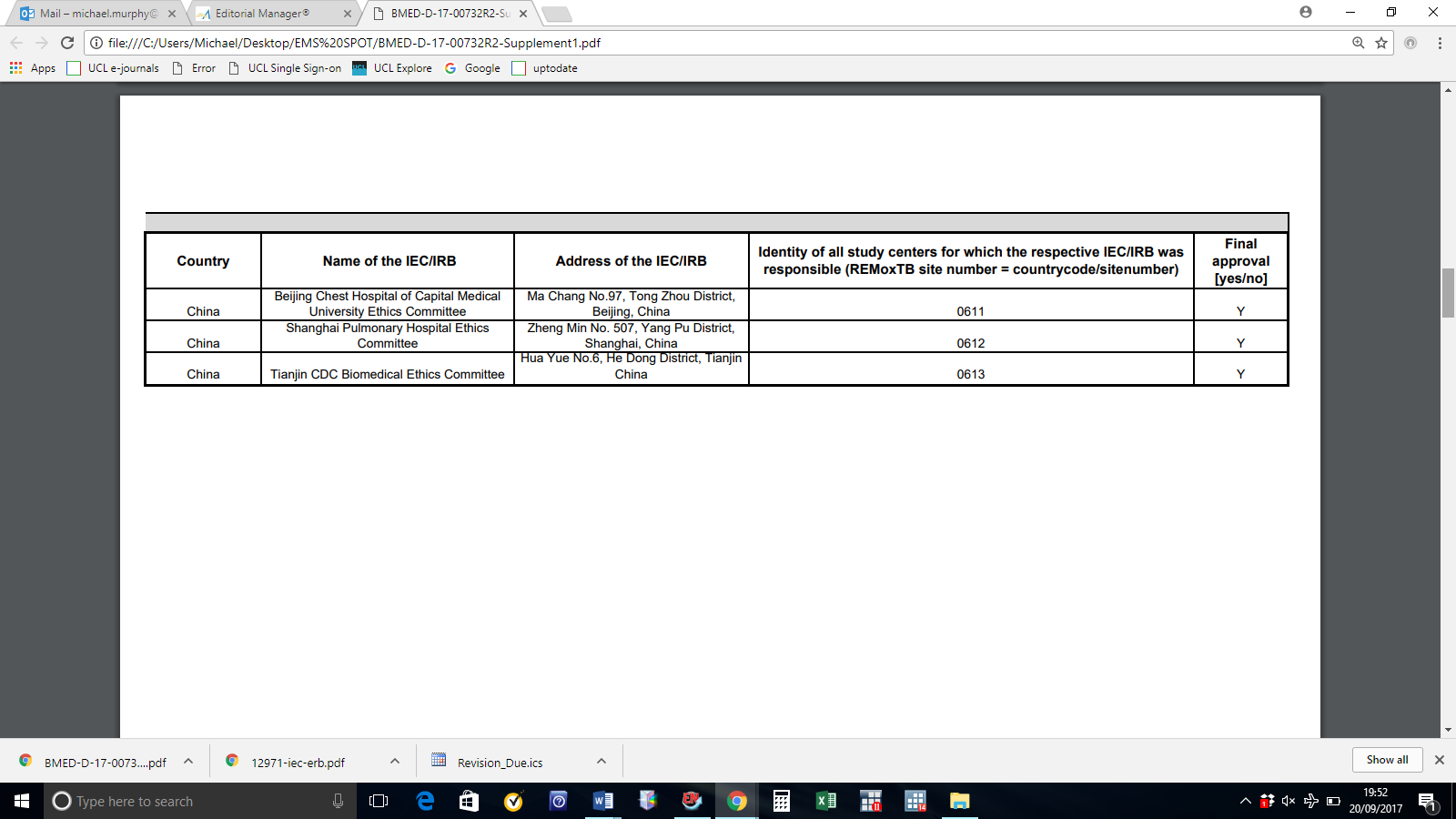


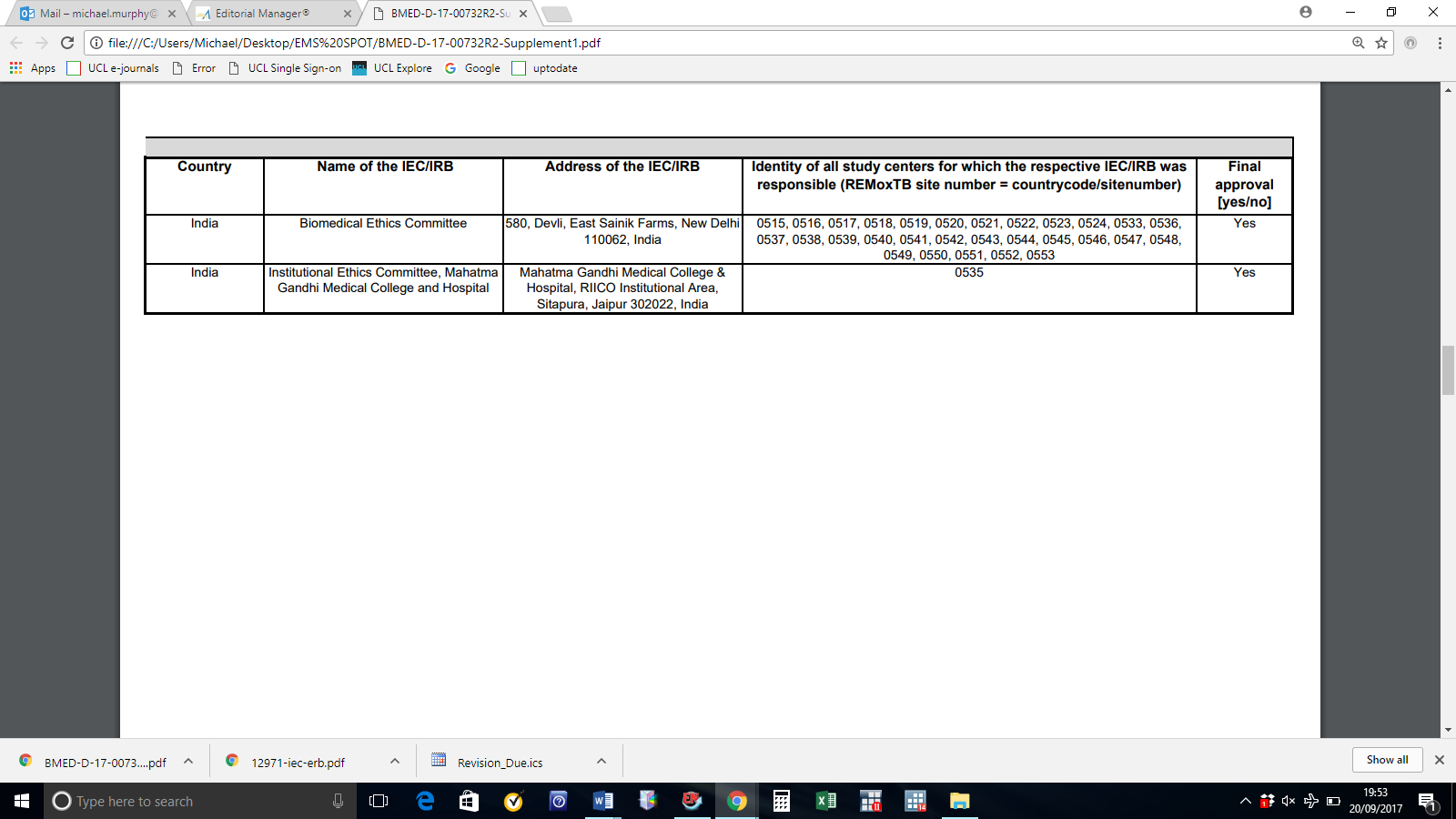


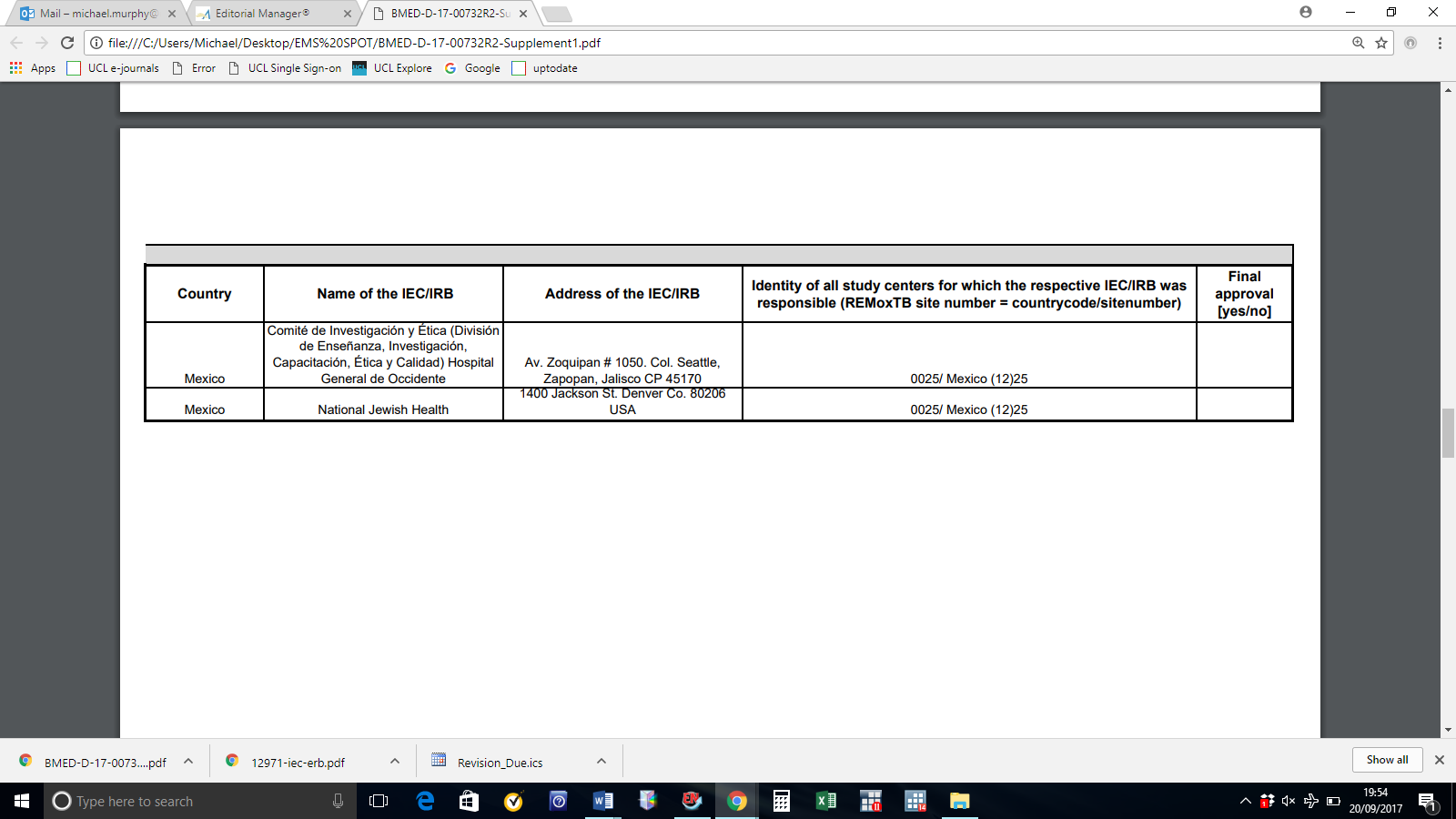


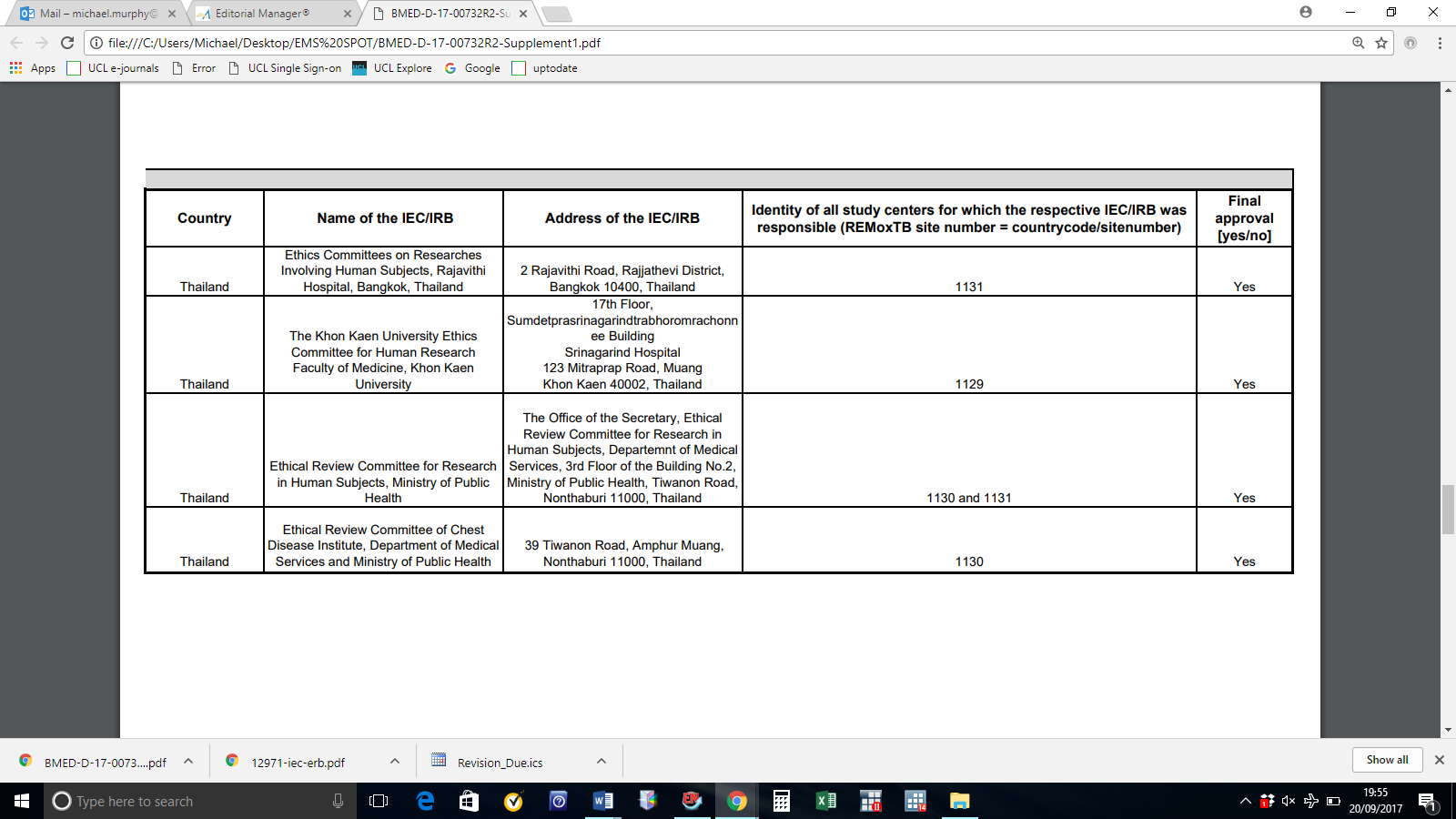


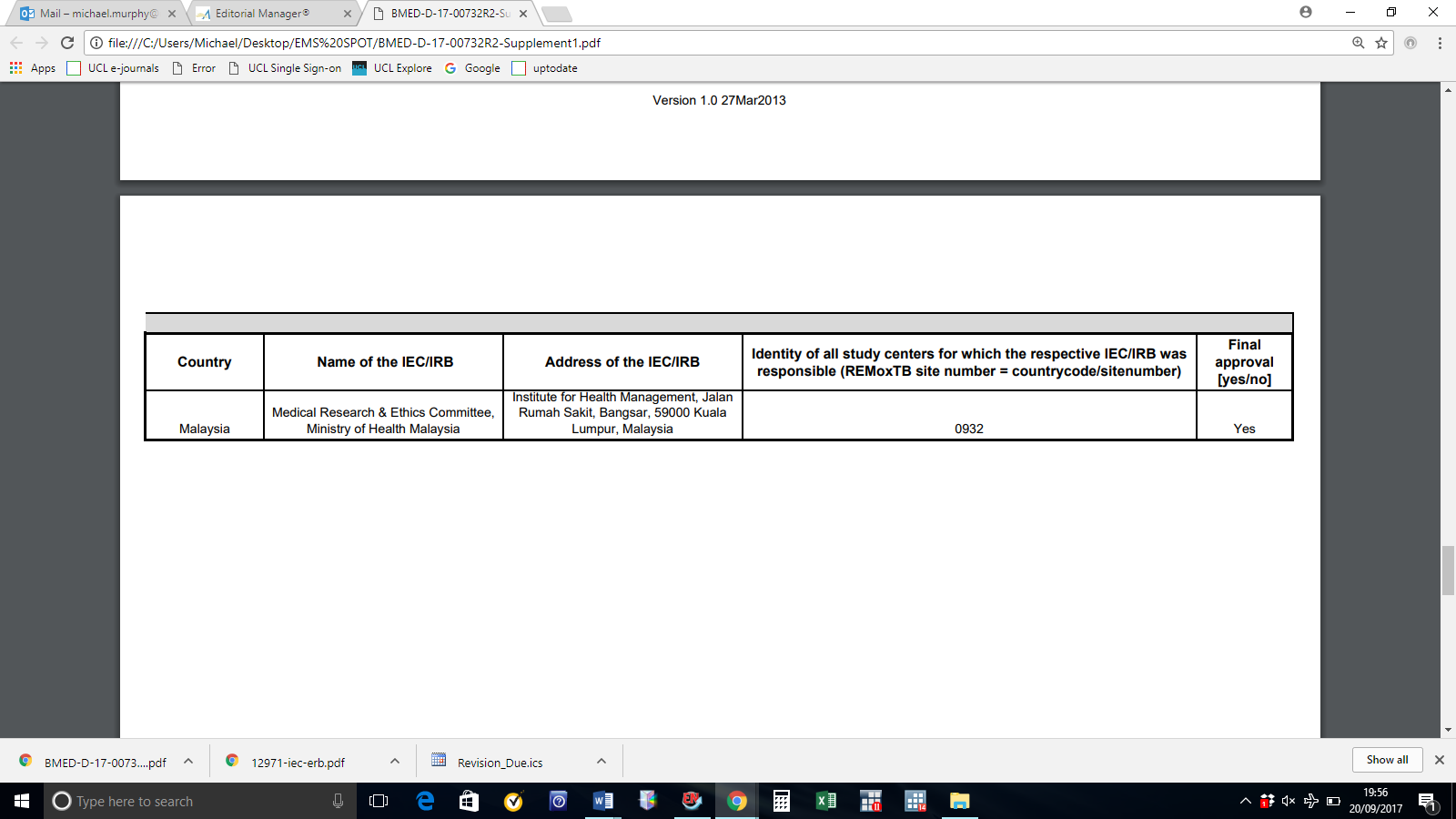

Supplement: Additional file 1: — List of ethics committee approving the REMoxTB study. (DOCX 893 kb) [file 12916_2017_947_MOESM1_ESM.docx]
